# 4-Chloro-*N*-(3-chloro­phen­yl)-2-methyl­benzene­sulfonamide

**DOI:** 10.1107/S1600536810022968

**Published:** 2010-06-18

**Authors:** B. Thimme Gowda, Sabine Foro, P. G. Nirmala, Hartmut Fuess

**Affiliations:** a Department of Chemistry, Mangalore University, Mangalagangotri 574 199, Mangalore, India; b Institute of Materials Science, Darmstadt University of Technology, Petersenstrasse 23, D-64287 Darmstadt, Germany

## Abstract

In the title compound, C_13_H_11_Cl_2_NO_2_S, the conformation of the N—H bond in the C—SO_2_—NH—C segment is *anti* to the *meta*-Cl atom on the aniline ring and *syn* to the *ortho*-methyl group on the sulfonyl­benzene ring. Furthermore, the torsion angle of the C—SO_2_—NH—C segment in the mol­ecule is 80.1 (3)°. The two benzene rings are tilted relative to each other by 70.9 (1)°. In the crystal, pairs of inter­molecular N—H⋯O hydrogen bonds link the mol­ecules *via* inversion-related dimers into infinite column-like chains.

## Related literature

For the preparation of the title compound, see: Savitha & Gowda (2006 ▶). For our studies of the effect of substituents on the structures of *N*-(ar­yl)aryl­sulfonamides, see: Gowda *et al.* (2008 ▶, 2009**a* ▶,b* ▶). For related structures, see: Gelbrich *et al.* (2007 ▶); Perlovich *et al.* (2006 ▶).
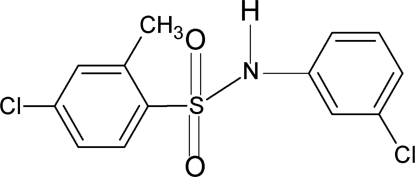


## Experimental

### 

#### Crystal data

C_13_H_11_Cl_2_NO_2_S
*M* *_r_* = 316.19
Monoclinic, 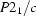

*a* = 7.9757 (7) Å
*b* = 11.3472 (8) Å
*c* = 15.569 (1) Å
β = 91.490 (8)°
*V* = 1408.55 (18) Å^3^
*Z* = 4
Mo *K*α radiation
μ = 0.61 mm^−1^
*T* = 299 K
0.36 × 0.28 × 0.04 mm

#### Data collection

Oxford Diffraction Xcalibur diffractometer with a Sapphire CCD detector
Absorption correction: multi-scan (*CrysAlis RED*; Oxford Diffraction, 2009 ▶) *T*_min_ = 0.812, *T*_max_ = 0.976
5849 measured reflections
2866 independent reflections
1853 reflections with *I* > 2σ(*I*)
*R*_int_ = 0.017

#### Refinement

*R*[*F* ^2^ > 2σ(*F* ^2^)] = 0.055
*wR*(*F* ^2^) = 0.161
*S* = 1.04
2866 reflections
176 parameters
1 restraint
H atoms treated by a mixture of independent and constrained refinement
Δρ_max_ = 0.43 e Å^−3^
Δρ_min_ = −0.52 e Å^−3^

### 

Data collection: *CrysAlis CCD* (Oxford Diffraction, 2009 ▶); cell refinement: *CrysAlis RED* (Oxford Diffraction, 2009 ▶); data reduction: *CrysAlis RED*; program(s) used to solve structure: *SHELXS97* (Sheldrick, 2008 ▶); program(s) used to refine structure: *SHELXL97* (Sheldrick, 2008 ▶); molecular graphics: *PLATON* (Spek, 2009 ▶); software used to prepare material for publication: *SHELXL97*.

## Supplementary Material

Crystal structure: contains datablocks I, global. DOI: 10.1107/S1600536810022968/vm2031sup1.cif

Structure factors: contains datablocks I. DOI: 10.1107/S1600536810022968/vm2031Isup2.hkl

Additional supplementary materials:  crystallographic information; 3D view; checkCIF report

## Figures and Tables

**Table 1:** Hydrogen-bond geometry (Å, °)

*D*—H⋯*A*	*D*—H	H⋯*A*	*D*⋯*A*	*D*—H⋯*A*
N1—H1*N*⋯O2^i^	0.85 (2)	2.08 (2)	2.926 (4)	175 (4)

Symmetry code: (i) 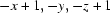.

**Fig. 1.**
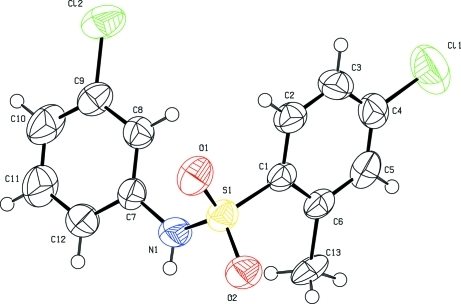
Molecular structure of (I), showing the atom labelling scheme and displacement ellipsoids are drawn at the 50% probability level.

**Fig. 2.**
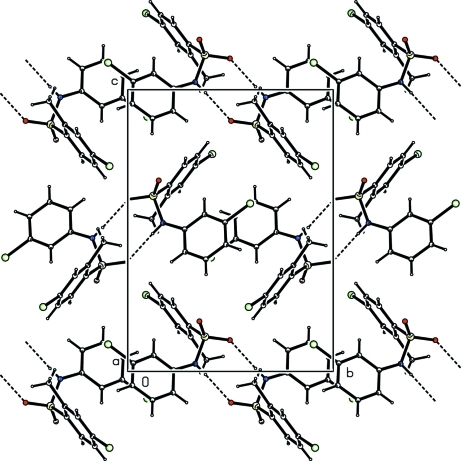
Molecular packing of (I) with hydrogen bonding shown as dashed lines.

## supplementary crystallographic information

### Comment

As part of a study of the substituent effects on the crystal structures of *N*-(aryl)-arylsulfonamides (Gowda *et al.*, 2008; Gowda *et al.*, 2009*a,b*), in the present work, the structure of 4-chloro-2-methyl-*N*-(3-chlorophenyl)benzenesulfonamide (I) has been determined (Fig. 1). The conformation of the N—H bond on the C—SO_2_—NH—C segment is *anti* to the *meta*-Cl on the aniline ring and *syn* to the *ortho*-methyl group in the sulfonyl benzene ring.

The torsion angle of the segment C—SO_2_—NH—C in (I) is 80.1 (3)°, compared to the values of -61.9 (4)° and 69.7 (4)° in the two independent molecules of 4-chloro-2-methyl-*N*-(phenyl)-benzenesulfonamide(II) (Gowda *et al.*, 2009*a*), 74.8 (4)° in 4-chloro-2-methyl-*N*-(2-chlorophenyl)-benzenesulfonamide (III) (Gowda *et al.*, 2009*b*) and -60.1 (2)° in *N*-(3-chlorophenyl)-benzenesulfonamide (IV)(Gowda *et al.*, 2008).

The sulfonyl and the aniline benzene rings in (I) are tilted relative to each other by 70.9 (1)°, compared to the values of 86.6 (2)° and 83.0 (2)° in the two independent molecules of (II), 45.5 (2)° in (III) and 65.4 (1)° in (IV).

The other bond parameters in (I) are similar to those observed in (II), (III), (IV) and other aryl sulfonamides (Perlovich *et al.*, 2006; Gelbrich *et al.*, 2007).

In the crystal structure, pairs of intermolecular N–H···O hydrogen bonds (Table 1) link the molecules into inversion-related dimers, which are further linked into infinite column like chains through C—H···π and C—Cl···π interactions. Part of the crystal structure is shown in Fig. 2.

### Experimental

The solution of *m*-chlorotoluene (10 cc) in chloroform (40 cc) was treated dropwise with chlorosulfonic acid (25 cc) at 0 ° C. After the initial evolution of hydrogen chloride subsided, the reaction mixture was brought to room temperature and poured into crushed ice in a beaker. The chloroform layer was separated, washed with cold water and allowed to evaporate slowly. The residual 2-methyl-4-chlorobenzenesulfonylchloride was treated with 3-chloroaniline in the stoichiometric ratio and boiled for ten minutes. The reaction mixture was then cooled to room temperature and added to ice cold water (100 cc). The resultant solid 4-chloro-2-methyl-*N*- (3-chlorophenyl)-benzenesulfonamide was filtered under suction and washed thoroughly with cold water. It was then recrystallized to constant melting point from dilute ethanol. The purity of the compound was checked and characterized by recording its infrared and NMR spectra (Savitha & Gowda, 2006).

The plate like colourless single crystals used in X-ray diffraction studies were grown in ethanolic solution by slow evaporation at room temperature.

### Refinement

The H atom of the NH group was located in a difference map and later restrained to N—H = 0.86 (2) Å. The other H atoms were positioned with idealized geometry using a riding model with C—H = 0.93–0.96 Å. All H atoms were refined with isotropic displacement parameters (set to 1.2 times of the *U*_eq_ of the parent atom).

### Crystal data

**Table:**

C_13_H_11_Cl_2_NO_2_S	*F*(000) = 648
*M**_r_* = 316.19	*D*_x_ = 1.491 Mg m^−^^3^
Monoclinic, *P*2_1_/*c*	Mo *K*α radiation, λ = 0.71073 Å
Hall symbol: -P 2ybc	Cell parameters from 2107 reflections
*a* = 7.9757 (7) Å	θ = 2.6–27.8°
*b* = 11.3472 (8) Å	µ = 0.61 mm^−^^1^
*c* = 15.569 (1) Å	*T* = 299 K
β = 91.490 (8)°	Plate, colourless
*V* = 1408.55 (18) Å^3^	0.36 × 0.28 × 0.04 mm
*Z* = 4	

### Data collection

**Table:**

Oxford Diffraction Xcalibur diffractometer with a Sapphire CCD detector	2866 independent reflections
Radiation source: fine-focus sealed tube	1853 reflections with *I* > 2σ(*I*)
graphite	*R*_int_ = 0.017
Rotation method data acquisition using ω and phi scans	θ_max_ = 26.4°, θ_min_ = 2.6°
Absorption correction: multi-scan (*CrysAlis RED*; Oxford Diffraction, 2009)	*h* = −9→7
*T*_min_ = 0.812, *T*_max_ = 0.976	*k* = −14→14
5849 measured reflections	*l* = −19→19

### Refinement

**Table:**

Refinement on *F*^2^	Primary atom site location: structure-invariant direct methods
Least-squares matrix: full	Secondary atom site location: difference Fourier map
*R*[*F*^2^ > 2σ(*F*^2^)] = 0.055	Hydrogen site location: inferred from neighbouring sites
*wR*(*F*^2^) = 0.161	H atoms treated by a mixture of independent and constrained refinement
*S* = 1.04	*w* = 1/[σ^2^(*F*_o_^2^) + (0.0763*P*)^2^ + 0.8836*P*] where *P* = (*F*_o_^2^ + 2*F*_c_^2^)/3
2866 reflections	(Δ/σ)_max_ = 0.001
176 parameters	Δρ_max_ = 0.43 e Å^−^^3^
1 restraint	Δρ_min_ = −0.52 e Å^−^^3^

### Special details

**Table:**

Experimental. CrysAlis RED (Oxford Diffraction, 2009) Empirical absorption correction using spherical harmonics, implemented in SCALE3 ABSPACK scaling algorithm.
Geometry. All e.s.d.'s (except the e.s.d. in the dihedral angle between two l.s. planes) are estimated using the full covariance matrix. The cell e.s.d.'s are taken into account individually in the estimation of e.s.d.'s in distances, angles and torsion angles; correlations between e.s.d.'s in cell parameters are only used when they are defined by crystal symmetry. An approximate (isotropic) treatment of cell e.s.d.'s is used for estimating e.s.d.'s involving l.s. planes.
Refinement. Refinement of *F*^2^ against ALL reflections. The weighted *R*-factor *wR* and goodness of fit *S* are based on *F*^2^, conventional *R*-factors *R* are based on *F*, with *F* set to zero for negative *F*^2^. The threshold expression of *F*^2^ > σ(*F*^2^) is used only for calculating *R*-factors(gt) *etc*. and is not relevant to the choice of reflections for refinement. *R*-factors based on *F*^2^ are statistically about twice as large as those based on *F*, and *R*- factors based on ALL data will be even larger.

### Fractional atomic coordinates and isotropic or equivalent isotropic displacement parameters (Å^2^)

**Table:**

	*x*	*y*	*z*	*U*_iso_*/*U*_eq_	
C1	0.3431 (4)	0.2031 (3)	0.66150 (19)	0.0451 (7)	
C2	0.3719 (5)	0.2761 (3)	0.7319 (2)	0.0571 (9)	
H2	0.4793	0.2818	0.7563	0.069*	
C3	0.2436 (6)	0.3398 (3)	0.7657 (2)	0.0642 (10)	
H3	0.2638	0.3897	0.8122	0.077*	
C4	0.0858 (5)	0.3293 (3)	0.7305 (2)	0.0617 (10)	
C5	0.0557 (5)	0.2571 (3)	0.6608 (3)	0.0630 (10)	
H5	−0.0530	0.2507	0.6383	0.076*	
C6	0.1829 (4)	0.1936 (3)	0.6234 (2)	0.0508 (8)	
C7	0.6346 (4)	0.2765 (3)	0.5046 (2)	0.0486 (8)	
C8	0.6426 (4)	0.3741 (3)	0.5575 (2)	0.0543 (8)	
H8	0.5940	0.3729	0.6111	0.065*	
C9	0.7240 (5)	0.4732 (3)	0.5292 (2)	0.0585 (9)	
C10	0.7951 (6)	0.4781 (4)	0.4506 (3)	0.0809 (13)	
H10	0.8492	0.5459	0.4325	0.097*	
C11	0.7847 (6)	0.3796 (4)	0.3983 (3)	0.0887 (15)	
H11	0.8319	0.3815	0.3444	0.106*	
C12	0.7061 (5)	0.2795 (4)	0.4249 (2)	0.0641 (10)	
H12	0.7007	0.2137	0.3893	0.077*	
C13	0.1457 (5)	0.1168 (3)	0.5412 (2)	0.0595 (10)	
H13A	0.1807	0.0371	0.5519	0.071*	
H13B	0.2060	0.1481	0.4937	0.071*	
H13C	0.0276	0.1182	0.5276	0.071*	
N1	0.5495 (4)	0.1719 (3)	0.5270 (2)	0.0640 (9)	
H1N	0.539 (5)	0.120 (3)	0.488 (2)	0.077*	
O1	0.6571 (3)	0.1510 (2)	0.67778 (17)	0.0697 (7)	
O2	0.4690 (3)	0.0031 (2)	0.61100 (16)	0.0660 (7)	
Cl1	−0.0771 (2)	0.41129 (13)	0.77137 (10)	0.1162 (6)	
Cl2	0.73479 (16)	0.59549 (9)	0.59594 (8)	0.0858 (4)	
S1	0.51639 (11)	0.12421 (7)	0.62307 (6)	0.0533 (3)	

### Atomic displacement parameters (Å^2^)

**Table:**

	*U*^11^	*U*^22^	*U*^33^	*U*^12^	*U*^13^	*U*^23^
C1	0.0512 (19)	0.0407 (16)	0.0432 (17)	−0.0108 (14)	0.0010 (14)	0.0041 (13)
C2	0.062 (2)	0.061 (2)	0.0488 (19)	−0.0148 (18)	−0.0021 (16)	−0.0027 (17)
C3	0.087 (3)	0.057 (2)	0.049 (2)	−0.011 (2)	0.012 (2)	−0.0088 (17)
C4	0.070 (3)	0.048 (2)	0.068 (2)	−0.0030 (18)	0.023 (2)	0.0086 (18)
C5	0.049 (2)	0.058 (2)	0.082 (3)	−0.0104 (18)	0.0034 (19)	0.012 (2)
C6	0.054 (2)	0.0417 (18)	0.057 (2)	−0.0135 (15)	0.0006 (16)	0.0043 (15)
C7	0.0451 (18)	0.0475 (18)	0.0533 (19)	−0.0033 (15)	0.0043 (15)	0.0037 (15)
C8	0.055 (2)	0.050 (2)	0.058 (2)	−0.0003 (16)	0.0070 (16)	0.0008 (16)
C9	0.059 (2)	0.0453 (19)	0.070 (2)	−0.0021 (17)	−0.0067 (18)	0.0079 (17)
C10	0.098 (3)	0.072 (3)	0.073 (3)	−0.031 (2)	0.002 (2)	0.020 (2)
C11	0.105 (4)	0.100 (4)	0.061 (3)	−0.034 (3)	0.021 (2)	0.010 (3)
C12	0.069 (2)	0.068 (2)	0.055 (2)	−0.010 (2)	0.0066 (19)	−0.0050 (18)
C13	0.056 (2)	0.058 (2)	0.063 (2)	−0.0245 (17)	−0.0249 (17)	−0.0032 (17)
N1	0.084 (2)	0.0510 (18)	0.0578 (19)	−0.0174 (16)	0.0191 (16)	−0.0079 (14)
O1	0.0540 (15)	0.0750 (18)	0.0794 (18)	−0.0031 (13)	−0.0104 (13)	0.0085 (14)
O2	0.0826 (19)	0.0420 (13)	0.0738 (17)	−0.0038 (12)	0.0094 (14)	0.0032 (11)
Cl1	0.1189 (12)	0.0990 (10)	0.1336 (12)	0.0235 (8)	0.0566 (10)	0.0030 (8)
Cl2	0.1005 (9)	0.0488 (6)	0.1077 (9)	−0.0067 (5)	−0.0032 (7)	−0.0073 (5)
S1	0.0557 (5)	0.0457 (5)	0.0584 (5)	−0.0046 (4)	0.0028 (4)	0.0040 (4)

### Geometric parameters (Å, °)

**Table:**

C1—C2	1.388 (4)	C8—H8	0.9300
C1—C6	1.399 (5)	C9—C10	1.362 (6)
C1—S1	1.764 (3)	C9—Cl2	1.734 (4)
C2—C3	1.369 (5)	C10—C11	1.384 (6)
C2—H2	0.9300	C10—H10	0.9300
C3—C4	1.365 (5)	C11—C12	1.366 (5)
C3—H3	0.9300	C11—H11	0.9300
C4—C5	1.375 (5)	C12—H12	0.9300
C4—Cl1	1.733 (4)	C13—H13A	0.9600
C5—C6	1.386 (5)	C13—H13B	0.9600
C5—H5	0.9300	C13—H13C	0.9600
C6—C13	1.571 (5)	N1—S1	1.619 (3)
C7—C12	1.380 (5)	N1—H1N	0.850 (18)
C7—C8	1.380 (5)	O1—S1	1.424 (3)
C7—N1	1.416 (4)	O2—S1	1.436 (2)
C8—C9	1.377 (5)		
			
C2—C1—C6	120.7 (3)	C8—C9—Cl2	118.5 (3)
C2—C1—S1	117.1 (3)	C9—C10—C11	118.4 (4)
C6—C1—S1	122.1 (2)	C9—C10—H10	120.8
C3—C2—C1	120.6 (3)	C11—C10—H10	120.8
C3—C2—H2	119.7	C12—C11—C10	121.0 (4)
C1—C2—H2	119.7	C12—C11—H11	119.5
C4—C3—C2	119.3 (3)	C10—C11—H11	119.5
C4—C3—H3	120.3	C11—C12—C7	119.7 (4)
C2—C3—H3	120.3	C11—C12—H12	120.1
C3—C4—C5	120.6 (4)	C7—C12—H12	120.1
C3—C4—Cl1	119.9 (3)	C6—C13—H13A	109.5
C5—C4—Cl1	119.5 (3)	C6—C13—H13B	109.5
C4—C5—C6	121.8 (4)	H13A—C13—H13B	109.5
C4—C5—H5	119.1	C6—C13—H13C	109.5
C6—C5—H5	119.1	H13A—C13—H13C	109.5
C5—C6—C1	116.9 (3)	H13B—C13—H13C	109.5
C5—C6—C13	120.4 (3)	C7—N1—S1	126.7 (3)
C1—C6—C13	122.7 (3)	C7—N1—H1N	116 (3)
C12—C7—C8	120.2 (3)	S1—N1—H1N	115 (3)
C12—C7—N1	117.0 (3)	O1—S1—O2	118.89 (17)
C8—C7—N1	122.8 (3)	O1—S1—N1	109.67 (17)
C9—C8—C7	118.7 (3)	O2—S1—N1	104.33 (16)
C9—C8—H8	120.7	O1—S1—C1	107.51 (16)
C7—C8—H8	120.7	O2—S1—C1	108.91 (15)
C10—C9—C8	122.1 (4)	N1—S1—C1	106.97 (16)
C10—C9—Cl2	119.4 (3)		
			
C6—C1—C2—C3	0.4 (5)	C8—C9—C10—C11	−0.2 (7)
S1—C1—C2—C3	−179.7 (3)	Cl2—C9—C10—C11	−179.9 (4)
C1—C2—C3—C4	1.2 (5)	C9—C10—C11—C12	−0.4 (7)
C2—C3—C4—C5	−1.0 (5)	C10—C11—C12—C7	0.5 (7)
C2—C3—C4—Cl1	−178.9 (3)	C8—C7—C12—C11	−0.1 (6)
C3—C4—C5—C6	−0.7 (6)	N1—C7—C12—C11	177.6 (4)
Cl1—C4—C5—C6	177.2 (3)	C12—C7—N1—S1	155.4 (3)
C4—C5—C6—C1	2.1 (5)	C8—C7—N1—S1	−27.0 (5)
C4—C5—C6—C13	−177.0 (3)	C7—N1—S1—O1	−36.2 (4)
C2—C1—C6—C5	−2.0 (5)	C7—N1—S1—O2	−164.6 (3)
S1—C1—C6—C5	178.1 (2)	C7—N1—S1—C1	80.1 (3)
C2—C1—C6—C13	177.2 (3)	C2—C1—S1—O1	2.0 (3)
S1—C1—C6—C13	−2.8 (4)	C6—C1—S1—O1	−178.1 (3)
C12—C7—C8—C9	−0.5 (5)	C2—C1—S1—O2	132.0 (3)
N1—C7—C8—C9	−178.1 (3)	C6—C1—S1—O2	−48.0 (3)
C7—C8—C9—C10	0.6 (6)	C2—C1—S1—N1	−115.7 (3)
C7—C8—C9—Cl2	−179.7 (3)	C6—C1—S1—N1	64.2 (3)

### Hydrogen-bond geometry (Å, °)

**Table:**

*D*—H···*A*	*D*—H	H···*A*	*D*···*A*	*D*—H···*A*
N1—H1N···O2^i^	0.85 (2)	2.08 (2)	2.926 (4)	175 (4)

Symmetry codes: (i) −*x*+1, −*y*, −*z*+1.
